# STATE-BASED ESTIMATES OF COGNITIVE DIFFICULTY AMONG MIDDLE EASTERN AND NORTH AFRICAN AMERICANS

**DOI:** 10.1093/geroni/igae098.1178

**Published:** 2024-12-31

**Authors:** Tiffany Kindratt, Alexandra Smith

**Affiliations:** The University of Texas Arlington, Arlington, Texas, United States; The University of Texas Arlington, Arlington, Texas, United States

## Abstract

Research on cognitive difficulty in the US focuses on racial/ethnic groups that comprise federal minimum reporting guidelines, including White, Black, Hispanic/Latino, Asian, American Indian/Alaskan Native (AI/AN), and Native Hawaiian/Other Pacific Islanders (NH/OPI). Research on Middle Eastern and North African (MENA) adults is limited, and no state-based estimates have been reported. Our objective was to estimate the prevalence of cognitive difficulty among MENA adults compared to other racial/ethnic groups in four states (California, New York, Michigan, and Texas) after adjusting for age and sex. We calculated MENA populations by state using race, ethnicity, ancestry and birthplace responses from the 2017-2021 American Community Survey. The four largest MENA populations were found in California (n=912,787), New York (n=341,361), Michigan (n=262,918) and Texas (n=258,395). Cognitive difficulty among MENA adults (yes/no; age>=45years) was compared to other racial/ethnic groups (e.g., White, Hispanic/Latino, Black). In California, the prevalence of cognitive difficulty was higher among MENA adults (6.96%) compared to White (4.80%), Hispanic/Latino (5.44%), and Asian (4.35%) adults. In New York, the prevalence was lower among MENA adults (3.96%) compared to Black (4.82%), Hispanic/Latino (7.96%), and AI/AN (8.94%) adults. In Michigan, the prevalence was lower among MENA (8.30%) compared to Black (11.32%) adults, but higher than White (6.25%) and Asian (3.28%) adults. In Texas, the prevalence was lower among MENA (4.44%) adults compared to Black (8.41%), Hispanic/Latino (6.28%), and Asian (8.41%) adults (all p’s<.05). This study provides an important first look at state-based estimates of cognitive difficulties among MENA adults living in California, New York, Michigan, and Texas.

